# Liquid level sensor based on dynamic Fabry–Perot interferometers in processed capillary fiber

**DOI:** 10.1038/s41598-021-82193-5

**Published:** 2021-02-04

**Authors:** Pablo Roldán-Varona, Rosa Ana Pérez-Herrera, Luis Rodríguez-Cobo, Luis Reyes-González, Manuel López-Amo, José Miguel López-Higuera

**Affiliations:** 1 Photonics Engineering Group, University of Cantabria, 39005 Santander, Spain; 2 Instituto de Investigación Sanitaria Valdecilla (IDIVAL), 39011 Santander, Spain; 3 CIBER-bbn, Instituto de Salud Carlos III, 28029 Madrid, Spain; 4 Dpto of Electrical, Electronic and Communication Engineering and Institute of Smart Cities (ISC), Public University of Navarra, 31006 Pamplona, Spain

**Keywords:** Fibre optics and optical communications, Laser material processing

## Abstract

In this work, a novel optical fiber sensor capable of measuring both the liquid level and its refractive index is designed, manufactured and demonstrated through simulations and experimentally. For this, a silica capillary hollow-core fiber is used. The fiber, with a sensing length of 1.55 mm, has been processed with a femtosecond laser, so that it incorporates four holes in its structure. In this way, the liquid enters the air core, and it is possible to perform the sensing through the Fabry–Perot cavities that the liquid generates. The detection mode is in reflection. With a resolution of 4 μm (liquid level), it is in the state of the art of this type of sensor. The system is designed so that in the future it will be capable of measuring the level of immiscible liquids, that is, liquids that form stratified layers. It can be useful to determine the presence of impurities in tanks.

Optical fiber sensors (OFSs) have traditionally been used in multiple application fields, such as biology, industry, communications, chemistry, or clinical procedures in medicine. The possibility of discriminating different physical, chemical or biological parameters in complex or inaccessible environments makes its presence very relevant today, with a great capacity of improvement. In recent years, what is known as lab-in-fiber (LIF) has begun to take on a notable importance. A LIF sensor is characterized by providing a wide functionality by integrating in relatively small dimensions a large number of sensor elements^1–4^. Typically, these are optical structures that allow small volumes (μL, nL) of biological fluids and liquids to be interrogated^1,3,5^. In an optical fiber, this type of optofluidic structures are favored by the presence of the fiber core as a waveguide that allows the different elements to be optimally interconnected.

Typically there are two options for sensing fluids located around the fiber: by guiding the light to the cladding^6,7^ (or by modifying the geometry of the fiber^8,9^), or by entering the fluid inside the fiber^1,3,10,11^. The latter notably improves sensitivity, and has been achieved through the manufacture of microchannels or microfluidic reservoirs^1^. However, it is also possible to use optical fibers whose characteristics allow to dynamically introduce fluids into the core, such as hollow-core fibers (HCFs). This type of fiber, which has been used mostly in applications such as fiber lasers^12^, or non-linear optics^13^, is also a very useful platform for use as a sensor^14,15^. There are multiple types of HCFs, among which are hollow-core photonic band gap fiber^16^, hole-assisted dual-core fiber^17^, two-core hollow eccentric fiber^18^, single hole twin eccentric cores fiber^19^, or silica capillary, among others. This last fiber type is cheaper than the rest of the fibers aforementioned and allows splicing without collapsing air regions.

Within the large number of parameters that can be measured, the detection of the liquid level with optical fibers is a really useful application in corrosive environments (such as car batteries or chemical processing), or with flammable liquids (such as fuel tanks). Table 1 lists the most relevant works in the literature regarding liquid level detection.

Although it is possible to use structures such as long period gratings (LPGs)^20^ or fiber Bragg gratings (FBGs)^21^ to perform the liquid level sensing, it is common to use HCFs to perform this type of sensing, due to the advantages that they present with regard to the handling of liquids, mentioned above. Photonic crystal fibers (PCFs) have been used^22^, as well as more special fibers based on reflection originated by Bragg resonance^23^, with sensitivities in both cases of 1.1 dB/mm (power variation in transmission). In recent years, silica tubes have been used, with different air core diameters, and sensing lengths between 1 and 2 cm. Likewise, the use of antiresonant (AR) mechanism^24,25^ for detection has been common in such works. Liu et al. developed in 2019 the first liquid level sensor whose resolution was below μm ($\sim 0.7\ \upmu$m), with a sensing length of 4.73 mm^26^. The sensor, also based on AR mechanisms, uses power variations in transmission for its detection.

**Table 1:** Comparative table of the liquid detection sensors in the literature.

Ref.	Optical fiber	Sensing length	Operation principle	Parameter	Sensitivity	Resolution^a^ (liq. level)	Detection mode
^27^	ø$75$μm silica capillary	18 mm	AR mechanism	Liq. level	0.4 dB/mm	25 μm	Transm.
^26^	ø30 μm silica capillary	4.73 mm	AR mechanism	Liq. level	0.014 dB/μm	0.71 μm	Transm.
^23^	Hollow core Bragg fiber	18 mm	Bragg reflection	Liq. level	1.1 dB/mm	9.1 μm	Transm.
^28^	MMF-HCF-FBG	18 mm	MZI	Liq. level	1.145 nm/mm	26.2 μm	Transm.
			FBG	Temperature	15 pm/°C		
^20^	SMF with	40 mm	LPG	Liq. level	4.8%/mm	33 μm	Transm.
	LPG		($\Lambda =400$μm)		(RI = 1.456)		
^21^	SMF with	24 mm	Etched	Liq. level	2.56 dB/mm	$3.9$μm	Transm.
	FBG		FBG				
^22^	Hollow-core	10 mm	AR mechanism	Liq. level	1.1 dB/mm	9.1 μm	Transm.
	PCF			Temperature	$-0.48\text { nm/}^\circ \text {C}$		
This work	ø$60\ \upmu$m silica	1.55 mm	Dynamic	Liq. level	$0.00113\text { nm}^{-1}/$μm	4 μm	Refl.
	capillary		FPs	SRI	($@ 1.3-1.4$)		

a A wavelength resolution of 30 pm and intensity resolution of 0.01 dB (typical detector) are considered as reference.

To the best of the author’s knowledge, this work contains the first liquid level sensor that features a reflection detection mode, and uses Fabry–Perot mechanisms to perform sensing. For this, a silica capillary is used. By creating four holes with a femtosecond laser, it is possible for the liquid to enter the air core. Although the sensing length is shorter than in other reported works, a resolution of 4 μm is achieved, and it allows simultaneously determining both the liquid level and its refractive index. However, it is important to note that the Fabry–Perot dual-cavity (air–liquid) concept is mentioned by Lee et al. in ^29^.

## Sensor structure and manufacturing parameters

**Figure 1:**
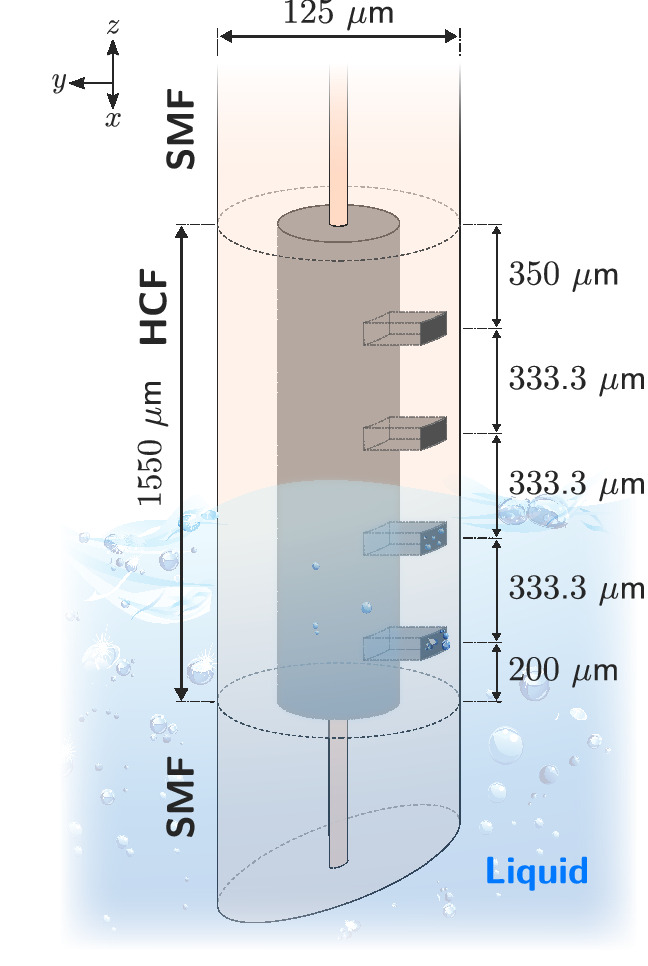
Schematic view of the manufactured sensor. It is based on a 1.55 mm long HCF with four holes fabricated by femtosecond laser ablation. This structure behaves as a liquid level sensor through the dynamic variation of the Fabry–Perot cavity length that forms the liquid within the fiber.

The designed sensor is schematically depicted in Fig. 1. It is based on a 1.55 mm long hollow-core fiber attached at its ends to individual sections of single-mode fiber (SMF). The HCF has been processed with a femtosecond laser in order to locate different holes that communicate the hollow-core with the surrounding medium. It should be noted that the HCF has a 60 μm diameter hollow-core.

**Figure 2:**
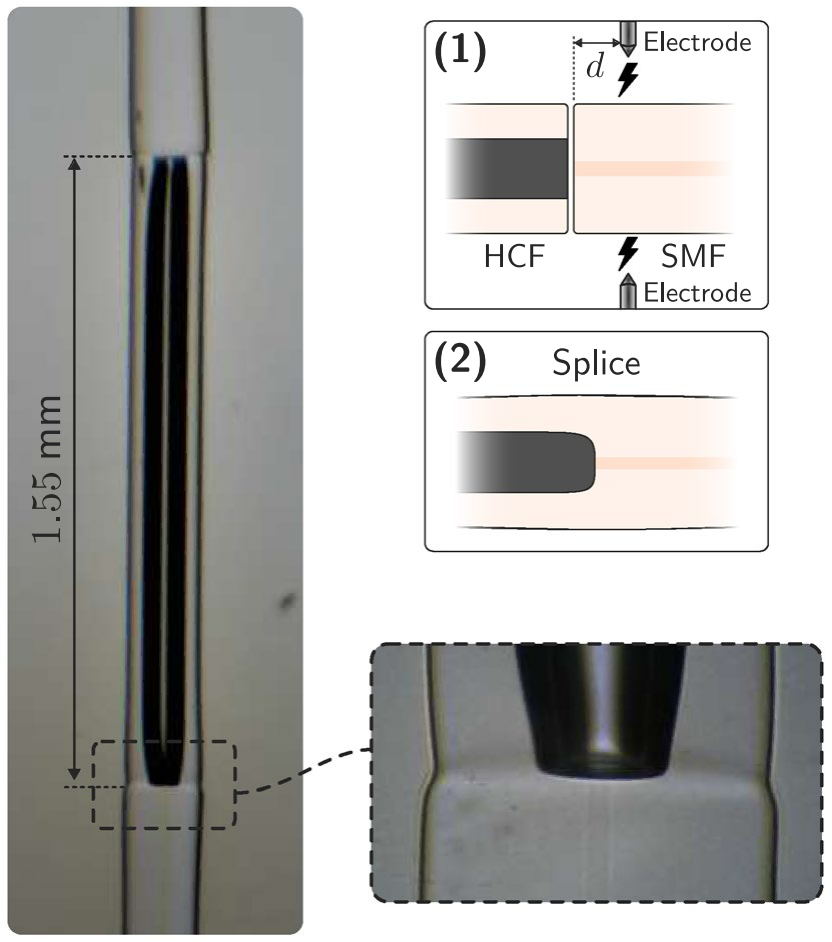
Microscope image of the HCF spliced to SMF sections (× 20 objective lens), with a zoom of the splice section (× 50 objective lens). A schematic of the HCF-SMF splice process is also depicted.

First, to perform the splice between HCF and SMF, the electric arc is not performed at the intersection, but slightly offset towards the side of the SMF ($d\simeq 100$μm in Fig.  2), and with the ends of both fibers practically together. Likewise, the electric arc is shorter (1 s) and less intense (18% of maximum current) than a normal splice, in order not to collapse the hollow-core. The result of the splice can be seen in Fig. 2. The contact surface of the hollow-core with the SMF core is flat, so losses are significantly reduced.

Next, the four holes that communicate the hollow-core with the surrounding medium are manufactured. These holes are located as shown in Fig. 1. It can be seen that the separation between each hole and the ends of the HCF is unique. This fact is important, as will be explained later in “Operating principle” section. For its manufacture, a commercial femtosecond Fiber Laser Chirp Pulse Amplifier (FLCPA) from CALMAR laser is used^30,31^. It operates at 1030 nm, and the pulses are tightly focused by a Mitutoyo objective lens ($\times 50$, NA$=0.42$). The sample, placed on an XYZ motor stage from Aerotech, is illuminated in transmission and displayed by a CMOS camera.

**Figure 3:**
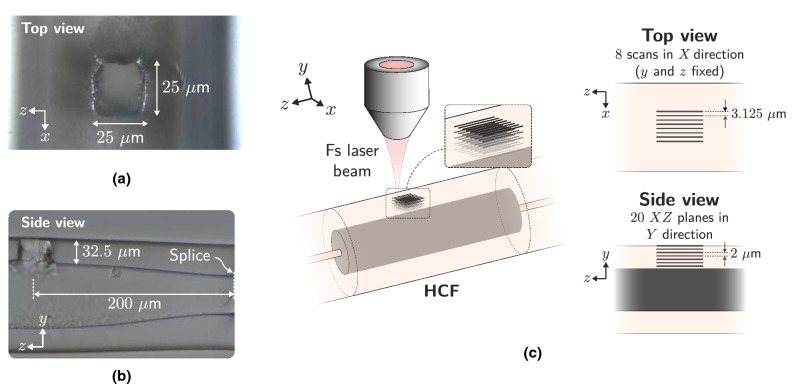
(**a**) Top view microscope image ($\times 50$ objective lens) of the processed HCF, with the square $25\times 25$μm hole. (**b**) Side view microscope image ($\times 20$ objective lens) of the hole corresponding to the final section of the processed HCF. (**c**) Schematic of the fs laser manufacturing process of the holes. 20 planes are made from the fiber surface to the hollow-core. Each plane corresponds to 8 laser scans of $25$μm in length.

The holes have dimensions of $25\times 25$μm (square shape) (Fig. 3a), going from the outside of the fiber to the hollow-core, so they have a depth of $\sim 32.5$μm (Fig. 3b). For its manufacture, a laser pulse energy ($E_p$) of $2.77$μJ is used, as well as a pulse repetition rate (PRR) of 60 kHz. The inscription process outlined in Fig. 3c is followed: the square shape of the hole is based on the inscription of 8 lines of $25$μm each, and $3.125$μm separated from each other. The writing speed is $v=2$μm/s, so 30, 000 pulses ($\text {PRR}/v$) are deposited for each μm of the fiber. Once the 8 lines have been inscribed, which define the plane located on the outer surface of the fiber, the laser inscription is repeated in a plane located 2 μm below, and so on until the inscription of 20 planes is made. The result obtained is shown in Fig. 3a and b. The separation between planes has been defined in such a way that the focal volume of the focused laser beam allows the material existing between both planes to be ablated. Given the objective lens used ($\text {NA}=0.42$), the Rayleigh length of the Gaussian beam focus (using the paraxial approximation^32^) is $y_0=2.7$μm. Consequently, the successive planes are arranged so that they are $2$μm apart, causing the focal volumes in both positions to be slightly overlapping.

### Operating principle

**Figure 4:**
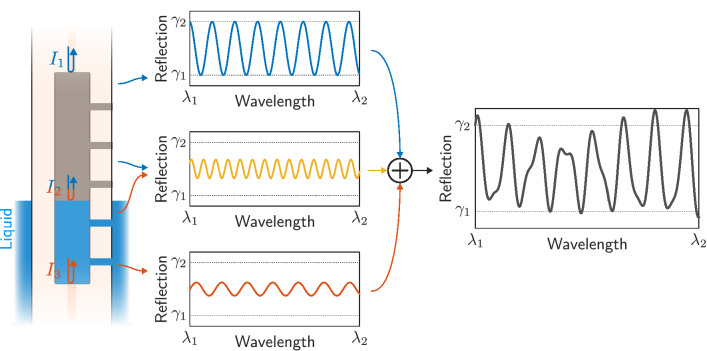
Schematic explanation of the manufactured sensor, with the interferometric contribution of the different Fabry–Perot cavities: air section, liquid section and both combined.

The operation principle of the manufactured sensor is based on interferometry. The 1.55 mm HCF acts as a single Fabry–Perot cavity when there is no liquid, or as a multi-cavity when liquid enters the fiber through the holes, as depicted in Fig. 4. In the latter case, the incident light that goes through the SMF core is reflected by the three surfaces present in the hollow-core fiber: core$_\text {SMF}-$air$_\text {HCF}$, air$_\text {HCF}-$liquid$_\text {HCF}$, and liquid$_\text {HCF}-$core$_\text {SMF}$. Consequently, there are three cavities, corresponding to the different combinations offered by these three existing surfaces in the HCF. These optical paths recombine in reflection, resulting in the interference pattern given by the next equation:

1 $$\begin{aligned} \begin{aligned} I&=I_1+I_2+I_3+\underbrace{2\sqrt{I_1I_2}\cos \left( \frac{2\pi n_{air}\cdot 2L_{air}}{\lambda }+\phi _{air}\right) }_{\text {Air} \, \text {section} \, \text {of} \, \text {HCF}} +\underbrace{2\sqrt{I_2I_3}\cos \left( \frac{2\pi n_{liquid}\cdot 2L_{liquid}}{\lambda }+\phi _{liquid}\right) }_{\text {Liquid section of HCF}}+ \\&+\underbrace{2\sqrt{I_1I_3}\cos \left( \frac{2\pi n_{HCF}\cdot 2L_{HCF}}{\lambda }+\phi _{HCF}\right) }_{\text {HCF} \text {section} \, (\text {air} \, \text {and} \, \text {liquid)}} +\underbrace{\sum _i\gamma _i\cdot \cos \left( \varphi _i\right) ,}_{\text {AR} \, \text {and} \, \text {other} \, \text {contributions}}\\&\text {where}\quad n_{air}=1,\quad L_{HCF}=1550\ \upmu \text {m},\quad L_{HCF}=L_{air}+L_{liquid},\quad n_{HCF}=\frac{t_{air}}{t_{air}+t_{liquid}}n_{air}+\frac{t_{liquid}}{t_{air}+t_{liquid}}n_{liquid},\\&\quad t_{air}=2\cdot L_{air}\frac{n_{air}}{c},\quad t_{liquid}=2\cdot L_{liquid}\frac{n_{liquid}}{c}. \end{aligned} \end{aligned}$$

$I_1$, $I_2$ and $I_3$ correspond to the light intensities reflected by the three mentioned surfaces, $\lambda$ is the wavelength of the incident light, $L_{air}$, $L_{liquid}$ and $L_{HCF}$ are the length of the cavities corresponding to the cavities formed in the HCF (air, liquid, and combination, respectively) ($2L_\text {\_}$ is the difference of optical paths), and $n_{air}$, $n_{liquid}$ and $n_{HCF}$ are the refractive indices of the cavities. $n_{HCF}$, effective refractive index of the cavity that forms the complete HCF (with air and liquid), must be computed taking into account the time that the light is in air ($t_{air}$) and liquid ($t_{liquid}$). *c* is the speed of light in vacuum.

The last term refers to intensity contributions whose importance is not relevant in this case. Fundamentally, it corresponds to the antiresonant mechanisms that take place in a structure such as SMF-HCF-SMF when hollow-core fiber has a length greater than what is known as the critical length ($L_c$)^24,25^. In this work, only Fabry–Perot mechanisms will be used.

It is important to note that the final section of the SMF is cleaved at an angle, as depicted in Fig. 1, thus avoiding unwanted reflections that could lead to other cavities. Consequently, the free spectral range (FSR) of the three dominant interference patterns corresponds to the following expression:

2 $$\begin{aligned} FSR_{i}=\frac{\lambda _m\lambda _{m+1}}{2\cdot n_i\cdot L_i},\quad \quad \text {where}\quad \quad \lambda _m=\frac{2\cdot n_i\cdot L_i}{m},\quad \quad \quad i \in \{air\text {, }liquid\text {, }HCF\},\ \ m \in {\mathbb {N}}. \end{aligned}$$

These FSR values, and therefore the refractive index of the liquid and its level (length of the cavities), can be obtained from the higher-order modes of the fast Fourier transform (FFT) of the spectral signal. To do this, it is essential to start from a known state. Hence, in the design, the holes have been made so that if there is liquid up to the level of each hole, the cavities that they create have unique lengths for each hole. However, HCF is typically considered to be liquid free in the initial state. The liquid filling process is noticeably slower than the spectra capture frequency, making it possible to track from the reference spectrum.

## Results and discussion

### Experimental setup

**Figure 5:**
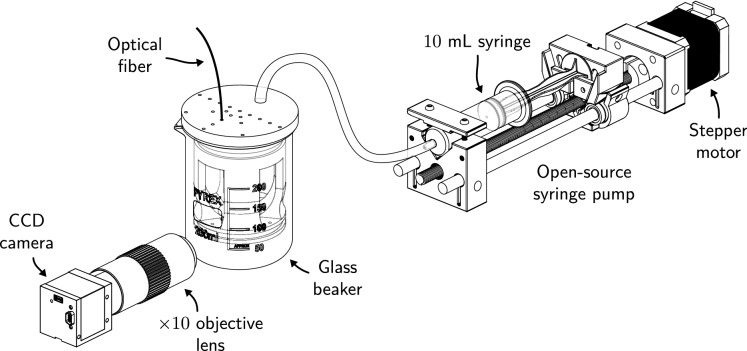
Depiction of the experimental setup used to regulate the liquid level in the hollow-core fiber.

The experimental measurement setup must be such as to ensure a resolution of μm in the liquid level of the HCF. For this, a syringe pump manufactured by 3D printing and controlled by an Arduino platform is used^33^. The vision system consists of a CCD camera and a $\times 10$ Mitutoyo objective lens. Figure 5 depicts the complete setup used. With the syringe used (10 mL), the minimum volume of liquid that the stepper motor can move is $1$μL, and with the beaker and the vision system used, that μL of liquid displacement in the syringe causes a movement of $0.65$μm in the liquid level of the beaker. Consequently, filling the 1.55 mm of the HCF implies a liquid displacement of 2.37 mL from the syringe. Moreover, the reflection spectra are obtained using a broadband light source (HP 83437A) and an optical spectrum analyzer (OSA) (Anritsu MS9740A, 30 pm resolution). The only consideration to take into account in choosing the light source is that it offers a power density such that the interfering signal reflected by the sensor is above the noise level, with a relatively high optical signal-to-noise ratio (OSNR). For this, it is important to highlight that, based on simulations carried out using the finite element bidirectional beam propagation method (bidirectional BPM) of RSoft software, there is a $\sim 11.6\%$ back-coupling efficiency from the silica capillary back to SMF (LP$_{01}$ guided mode).

### Experimental and simulated results

As mentioned above, the designed and manufactured sensor allows not only to determine the liquid level in the structure, but also the refractive index of the liquid. Consequently, the experimental results carried out, which are shown below, comprise two large groups. First, for different water levels, the real liquid level has been compared with the estimated one. Second, both the liquid level and the refractive index have been determined for different liquids. In both cases, simulations have been carried out using bidirectional BPM of RSoft software. The sensor does not measure temperature and, therefore, is not able to compensate for deviations in its value. Consequently, the experiments must be carried out at a constant temperature from the first reference measurement.

**Figure 6:**
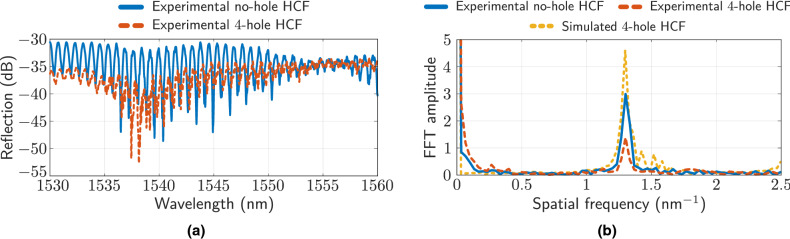
(**a**) Reflection spectra of the no-hole HCF (Fig. 2), and the 4-hole HCF (Fig. 3b). (**b**) FFT amplitude from the spectra of (**a**), as well as the one corresponding to the simulated structure.

The spectral characterization of the manufactured structure is presented in Fig. 6. In Fig. 6a, the spectral response of the HCF without holes is depicted (Fig. 2), as well as the response when HCF has been processed with the fs laser (four holes inscribed). It can be seen that making holes causes an increase in losses, and a reduction in the amplitude of the interference pattern. Likewise, in both cases, in addition to the Fabry–Perot cavity corresponding to the HCF, the presence of an interferometric contribution with a lower frequency component, associated with AR mechanisms, is deduced. Figure 6b depicts the FFT amplitude of the spectra in Fig. 6a, as well as the one corresponding to the simulation of the structure. The realization of the holes does not modify the spatial frequency but only the amplitude of the spectral contribution of the Fabry–Perot cavity. The dominant spatial frequency has a value of $1.3\text { nm}^{-1}$, which corresponds to a cavity length of $\sim 1550$μm (real HCF length).

**Figure 7:**
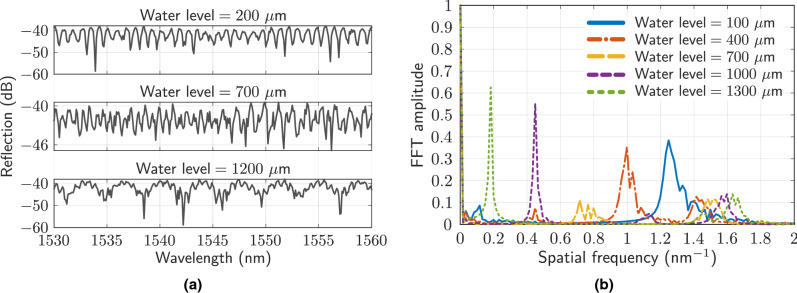
(**a**) Examples of measured reflection spectra for water levels of $200\ \upmu$m, $700\ \upmu$m and $1200\ \upmu$m, respectively.(**b**) FFT amplitude of measured spectra corresponding to water levels of $100\ \upmu$m, $400\ \upmu$m, $700\ \upmu$m, $1000\ \upmu$m and $1300\ \upmu$m.

**Figure 8:**
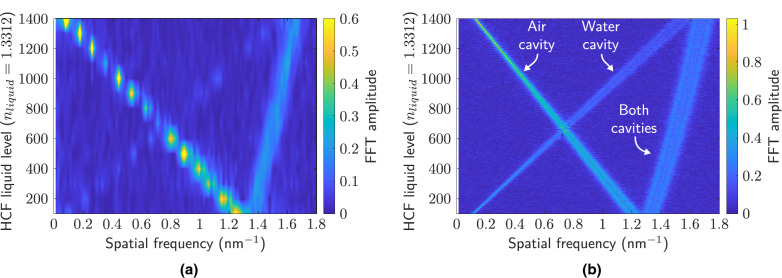
(**a**) Experimental FFT amplitude measured for water levels in the HCF between 100 and 1400 $\upmu$m, with a spacing of 100 $\upmu$m. (**b**) Simulated FFT amplitude for water levels in the HCF between 100 and 1400 $\upmu$m.

Figure 7 depicts the experimental spectra for different levels of liquid in the HCF (200, 700 and $1200$μm) (Fig. 7a), as well as the resulting FFT spectra for liquid levels of 100, 400, 700, 1000 and $1300$μm (Fig. 7b). The liquid is water, with a refractive index of 1.318. It is important to note that during the filling of the HCF, it is necessary to wait approximately $\sim 90$ seconds ($25\times 25$μm hole) in order to stabilize the liquid and thus obtain a stable spectral pattern. Larger holes cause faster filling^34^, but greater degradation in the amplitude of the interference spectral signal. Moreover, a filled contour plot of the spatial frequency corresponding to liquid levels between 100 and $1400$μm measured experimentally (Fig. 8a) or by simulation (Fig. 8b) is depicted.

In the FFT spectra, the presence of three large contributions can be observed, corresponding to the air, water, and joint cavities (complete HCF), as previously indicated in Eq. (1). In this work, unlike those indicated in the introduction, the spatial frequency shift is evaluated, and not the power variation due to the uncertainty that this parameter may present. The sensitivities determined for the experimental and simulation measurements are: $-8.89\cdot 10^{-4}\text { nm}^{-1}/\upmu \text {m}$ and $-8.19\cdot 10^{-4}\text { nm}^{-1}/\upmu \text {m}$ (air cavity), $11.3\cdot 10^{-4}\text { nm}^{-1}/\upmu \text {m}$ and $10.9\cdot 10^{-4}\text { nm}^{-1}/\upmu \text {m}$ (water cavity), and $2.28\cdot 10^{-4}\text { nm}^{-1}/\upmu \text {m}$ and $2.75\cdot 10^{-4}\text { nm}^{-1}/\upmu \text {m}$ (both cavities), respectively.

**Figure 9:**
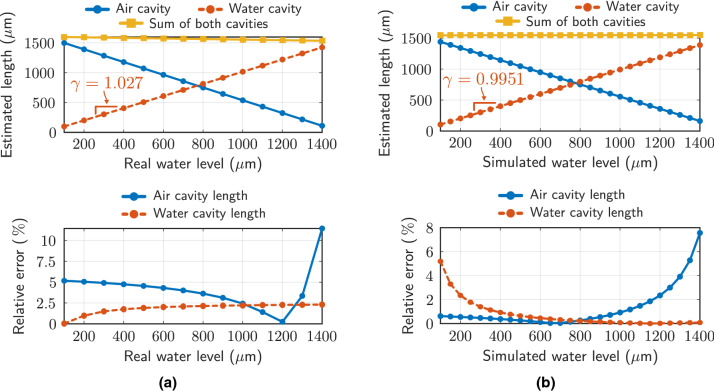
(**a**) Estimated cavity length (air and water) and relative error for different real water levels. (**b**) Estimated cavity length (air and water) and relative error for different simulated water levels.

From Eqs. (1) and (2), it is possible to determine the length of the cavity in each case. If the refractive index of the liquid is known, as in this case, the lengths of the air and liquid cavities can be obtained directly. In Fig. 9, the cavity lengths extracted from the spatial frequency are depicted, as well as the relative error committed, both for the experimental and simulation results. The slope corresponding to the real-estimated water level curve takes values $\gamma =1.027$ (experimental) and $\gamma =0.9951$ (simulation). Likewise, the relative error made in estimating the liquid level in experimental measurements is always less than $2.5\%$, so the results can be considered correct. The resolution, in this case, depends exclusively on the OSA, since the span and the number of points in the measurements determine the frequency spacing in the FFT spectrum. For the configuration used, the frequency resolution obtained is $0.00333\text { nm}^{-1}$, which corresponds to a resolution of $4\ \upmu$m in the liquid level. Although the spectral resolution of the OSA is the most important contribution regarding the calculation of the detection limit (DL)^35^, two types of noise contributions have to be taken into account^36^: amplitude noise (shot and thermal noise of the photodetector, and light source relative intensity noise), and spectral noise (thermal variation, and thermal-induced fluctuations because of the liquid in the capillary fiber), the second being the most important in this case. Considering water as a liquid, as well as a ${\mathcal {N}}(25,9)$ temperature distribution during the measurements, the DL obtained is $4.42\ \upmu$m (compared to the value of $2.95\ \upmu$m in the absence of noise). On the other hand, sensor repeatability is adequate. The relative error made between the values obtained by increasing and decreasing the liquid level, as well as by complete liquid removal and re-injection is less than $2\%$, with respect to the values in Fig. 9a.

**Figure 10:**
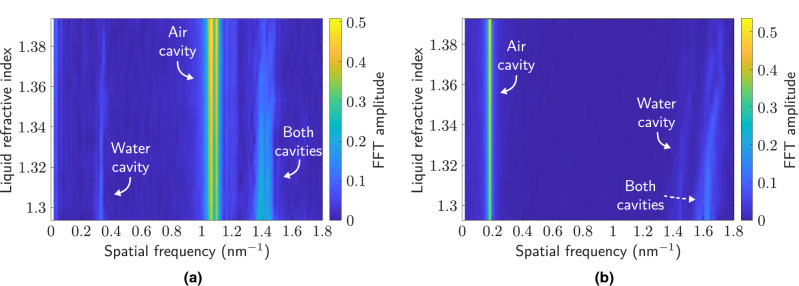
(**a**) Experimental FFT amplitude measured for different liquids in the HCF with a liquid level of $300\ \upmu$m. (**b**) Experimental FFT amplitude measured for different liquids in the HCF with a liquid level of $1300\ \upmu$m.

Subsequently, experimental measurements have been carried out with Cargille liquids of refractive indices between 1.3 and 1.4 (increment of 0.01), in order to determine both parameters (liquid level and RI) simultaneously. The liquid levels where the measurements have been made are 300 and $1300\ \upmu$m. The resulting FFT spectra for those liquid levels are depicted in Fig. 10.

It can be seen that, in both cases, the frequency corresponding to the air cavity remains fixed. The change of liquid in the cavity only affects the two remaining contributions. For detection, the air cavity length is first determined from its spatial frequency. Subsequently, the length of the liquid cavity is deduced by knowing the HCF length. For the cases presented, liquid cavity lengths of $287.25\ \upmu$m (it should be $300\ \upmu$m) and $1327.29\ \upmu$m (it should be $1300\ \upmu$m) have been determined, which offer relative errors of $4.25\%$ and $2.1\%$, respectively.

**Figure 11:**
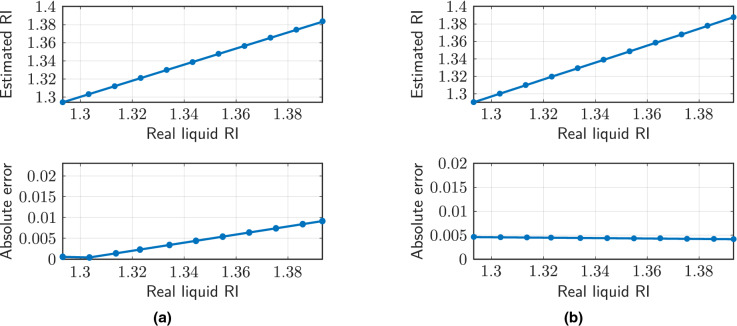
(**a**) Estimated refractive index of the HCF liquid whose level is at $300\ \upmu$m. (**b**) Estimated refractive index of the HCF liquid whose level is at $1300\ \upmu$m.

Once the length of the liquid cavity is known, it is possible to extract the refractive index of the cavity from the corresponding spatial frequency (Eq. 2). Figure 11 depicts the estimated refractive index, as well as the absolute error made, for the two cases presented in Fig. 10. The sensitivities of the spatial frequency corresponding to the liquid cavity are $0.2296\text { nm}^{-1}/$refractive index unit (RIU) (liquid level of $300\ \upmu$m) and $1.1099\text { nm}^{-1}/$RIU ($1300\ \upmu$m). The absolute error made is always lower than the order of the measurement step, so the sensor can be valid to offer an indicative measure of the liquid whose level is being evaluated. In any case, the precision in detecting the liquid level or the refractive index will be significantly higher when the other parameter is known. In this case, the worst scenario has been presented, in which none of the parameters are known.

## Conclusions

In this work, a novel sensor to evaluate the liquid level and its refractive index is designed, manufactured and experimentally demonstrated. The sensor is based on a 1.55 mm long silica capillary hollow-core fiber (ø$60\ \upmu$m core). This sensor section has been processed with a femtosecond laser in order to make four holes, allowing the liquid to enter the air core of the HCF. In this way, the sensitivity with respect to previous works is maximized, and dynamic Fabry–Perot mechanisms can be used to perform the sensing in reflection, something unprecedented in liquid level sensing. With a liquid level resolution of $4\ \upmu$m, the sensitivity obtained experimentally is $11.3\cdot 10^{-4}\text { nm}^{-1}/\upmu$m (spatial frequency shift), and its ability to measure liquids whose refractive index is between 1.3 and 1.4 has been verified. Likewise, it has been shown that the simulations agree with what was measured.

## References

[1] Haque M, Lee KKC, Ho S, Fernandes LA, Herman PR (2014). Chemical-assisted femtosecond laser writing of lab-in-fibers. Lab. Chip..

[2] Roldán-Varona P, Rodríguez-Cobo L, López-Higuera JM (2020). Reflection-based lab-in-fiber sensor integrated in a surgical needle for biomedical applications. Opt. Lett..

[3] Siyu E (2020). Two-channel surface plasmon resonance sensor for simultaneous measurement of seawater salinity and temperature. IEEE Trans. Instrum. Meas..

[4] Tian F, Sukhishvili S, Du H (2014). Photonic crystal fiber as a lab-in-fiber optofluidic platform. Springer Ser. Surf. Sci..

[5] Liao, C. *et al.* Femtosecond laser micromachining of microfluidic fiber sensors. In *2016 15th International Conference on Optical Communications and Networks (ICOCN)*. 10.1109/icocn.2016.7875603 (IEEE, 2016).

[6] Theodosiou A, Ioannou A, Kalli K (2019). All-in-fiber cladding interferometric and Bragg grating components made via plane-by-plane femtosecond laser inscription. J. Lightw. Technol..

[7] Roldán-Varona P, Pallarés-Aldeiturriaga D, Rodríguez-Cobo L, López-Higuera J (2020). All-in-fiber multiscan Mach–Zehnder interferometer assisted by core FBG for simultaneous multi-parameter sensing. Opt. Laser Technol..

[8] Galarza M, Perez-Herrera RA, Leandro D, Judez A, López-Amo M (2020). Spatial-frequency multiplexing of high-sensitivity liquid level sensors based on multimode interference micro-fibers. Sens. Actuators A..

[9] Mas S, Marti J, Palaci J (2015). Biconical tapered fibers manipulation for refractive index and strain sensing applications. IEEE Sens. J..

[10] Lomer M, Quintela A, López-Amo M, Zubia J, López-Higuera JM (2007). A quasi-distributed level sensor based on a bent side-polished plastic optical fibre cable. Meas. Sci. Technol..

[11] Liao C (2013). Tunable phase-shifted fiber Bragg grating based on femtosecond laser fabricated in-grating bubble. Opt. Lett..

[12] Xu M, Yu F, Knight J (2017). Mid-infrared 1W hollow-core fiber gas laser source. Opt. Lett..

[13] Millot G (2015). Frequency-agile dual-comb spectroscopy. Nat. Photon..

[14] Pérez-Herrera, R. A. *et al.* Multiplexing optical fiber Fabry–Perot interferometers based on air-microcavities. In Kalli, K., Brambilla, G. & O’Keeffe, S. O. (eds.) *Seventh European Workshop on Optical Fibre Sensors*, 10.1117/12.2540150 (SPIE, 2019).

[15] Pinto A, Baptista J, Santos J, Lopez-Amo M, Frazão O (2012). Micro-displacement sensor based on a hollow-core photonic crystal fiber. Sensors.

[16] Zhang Z (2018). Measurement of high pressure and high temperature using a dual-cavity Fabry–Perot interferometer created in cascade hollow-core fibers. Opt. Lett..

[17] Yang J (2018). In-fiber Mach–Zehnder interferometer with piecewise interference spectrum based on hole-assisted dual-core fiber for refractive index sensing. Opt. Express.

[18] Yuan T (2015). Long period fiber grating in two-core hollow eccentric fiber. Opt. Express.

[19] Ni W (2018). Simultaneous implementation of enhanced resolution and large dynamic range for fiber temperature sensing based on different optical transmission mechanisms. Opt. Express.

[20] Khaliq S, James SW, Tatam RP (2001). Fiber-optic liquid-level sensor using a long-period grating. Opt. Lett..

[21] Yun B, Chen N, Cui Y (2007). Highly sensitive liquid-level sensor based on etched fiber Bragg grating. IEEE Photon. Technol. Lett..

[22] Liu S (2013). Anti-resonant reflecting guidance in alcohol-filled hollow core photonic crystal fiber for sensing applications. Opt. Express.

[23] Wang Y, Yan G, Lian Z, Wu C, He S (2018). Liquid-level sensing based on a hollow core Bragg fiber. Opt. Express.

[24] Zhang X (2018). Transition of Fabry–Perot and antiresonant mechanisms via a SMF-capillary-SMF structure. Opt. Lett..

[25] Sun W (2020). Comparative study on transmission mechanisms in a SMF-capillary-SMF structure. J. Lightw. Technol..

[26] Liu D (2019). Sub-micrometer resolution liquid level sensor based on a hollow core fiber structure. Opt. Lett..

[27] Liu S, Tian J, Liu N, Xia J, Lu P (2016). Temperature insensitive liquid level sensor based on antiresonant reflecting guidance in silica tube. J. Lightw. Technol..

[28] Zhang Y (2018). High sensitivity optical fiber liquid level sensor based on a compact MMF-HCF-FBG structure. Meas. Sci. Technol..

[29] Lee C-L, Ho H-Y, Gu J-H, Yeh T-Y, Tseng C-H (2015). Dual hollow core fiber-based Fabry–Perot interferometer for measuring the thermo-optic coefficients of liquids. Opt. Lett..

[30] Gattass RR, Mazur E (2008). Femtosecond laser micromachining in transparent materials. Nat. Photon..

[31] Itoh K, Watanabe W, Nolte S, Schaffer CB (2006). Ultrafast processes for bulk modification of transparent materials. MRS Bull..

[32] Thomas J (2012). Femtosecond pulse written fiber gratings: A new avenue to integrated fiber technology. Laser Photon. Rev..

[33] Samokhin AS (2020). Syringe pump created using 3D printing technology and arduino platform. J. Anal. Chem..

[34] Liao CR, Hu T, Wang DN (2012). Optical fiber Fabry–Perot interferometer cavity fabricated by femtosecond laser micromachining and fusion splicing for refractive index sensing. Opt. Express.

[35] Loock H-P, Wentzell PD (2012). Detection limits of chemical sensors: Applications and misapplications. Sens. Actuators B..

[36] White IM, Fan X (2008). On the performance quantification of resonant refractive index sensors. Opt. Express.

